# Supplementary material for: Content-rich biological network constructed by mining PubMed abstracts
Source: BMC Bioinformatics. 2004 Oct 8;5:147. doi: 10.1186/1471-2105-5-147 (PMC528731; doi:10.1186/1471-2105-5-147)
Supplement: Additional File 2 — The original results of the above study (non-essential files are deleted to keep the file size under the limit set by BMC bioinformatics). [file 1471-2105-5-147-S2.bz2 › chilibotAdditionalFile2/dip10/386ID10318834E9856/html/EPO_EPOR.html]

 


 **EPO** and **EPOR** 
  
Found 716 abstracts in PubMed, retrieved 10.  
 

 What does Google say? 
 PDF only 
| .edu only 

---

**Interactive relationship** (e.g. stimulation, inhibition, etc)

**Neutral relationship**- Cytoprotection by  **EPO**  is initiated by the activation of the  **EPO**  receptor  **EPOR**  and subsequent signal transduction pathways that originate with the Janus tyrosine kinase 2 Jak2 protein.  Ref: 12769640 Curr Drug Targets Cardiovasc Haematol Disord, 2003
- Erythropoietin  [ **EPO** ]   **EPO**  regulates mammalian erythropoiesis by binding to its transmembrane receptor  **EPOR** .  Ref: 12878211 Biochem Biophys Res Commun, 2003
- These findings indicate the expression of multiple  **EPOR**  isoforms in human cancer cells that may modulate the cellular effects of recombinant human  **EPO**  or  **EPO**   **EPOR**  antagonists.  Ref: 12878211 Biochem Biophys Res Commun, 2003
- Signals provided by the  **Epo**  receptor  **EpoR**  are required for erythroid development beyond the CFUe stage, and are propagated via the  **EpoR**  tethered Janus kinase, JAK2.  Ref: 12869513 BloodBlood, 2003
- Presently, the activities of a PY null  **EpoR**  HM form in primary progenitor cells from knock in mice were further assessed using optimized  **Epo**  dose dependent proliferation, survival and differentiation assays.  Ref: 12869513 BloodBlood, 2003
- Strong evidence indicates that erythropoietin  [ **EPO** ]   **Epo**  is eliminated via  **Epo**  receptors  **EpoR** .  Ref: 12750427 J Pharmacol Exp Ther< MedlineTA>J Pharmacol Exp Ther, 2003

**Non-interactive relationship** (e.g. studied together, co-existance, homology, etc.)

- It has previously been demonstrated that mutations in the 232 WSAWS 236 motif in the erythropoietin  [ **EPO** ]  receptor  **EPOR**  can result in strongly inhibited surface expression, due to defective intracellular transport Hilton, D.  Ref: 12859190 Biochemistry, 2003
- As compared to the wt  **EpoR** ,  **EpoR**  HM activity was compromised several fold in each context when  **Epo**  was limited to physiological concentrations.  Ref: 12869513 BloodBlood, 2003
- Furthermore, administration of  **EPO**   **EPOR**  antagonists delayed the growth of uterine, ovarian, and mammary carcinoma cells in experimental animal models.  Ref: 12878211 Biochem Biophys Res Commun, 2003
- Using immunohistochemistry, we examined the expression of  **Epo** ,  **EpoR** , p16, hypoxia inducible factor HIF 1alpha, and bcl 2 in benign and dysplastic cervical squamous epithelia and invasive squamous cell carcinomas ISCCs.  Ref: 12759237 Am J Pathol, 2003
- Assuming that  **Epo**  s elimination largely depends not only on erythropoietic  **EpoR** , but also non hematopoietic  **EpoR** , this work shows a significant difference in the relative proportions of the two  **EpoR**  populations in lamb and adult sheep.  Ref: 12750427 J Pharmacol Exp Ther< MedlineTA>J Pharmacol Exp Ther, 2003
- Acting via its receptor  **EpoR** ,  **Epo**  up regulates bcl 2 and inhibits apoptosis of erythroid cells and rescues neurons from hypoxic damage.  Ref: 12759237 Am J Pathol, 2003
- The proliferation and survival of hematopoietic cells is strictly regulated by cytokine growth factors that act through receptors of the Type I cytokine receptor family, including erythropoietin  [ **EPO** ]   **Epo**  and its receptor,  **EpoR** .  Ref: 12746455 J Biol Chem< MedlineTA>J Biol Chem, 2003
- **Epo**  s elimination kinetics was studied using a very sensitive tracer interaction method TIM before and after chemical ablation of BM as an indirect way of evaluating the  **EpoR**  through an assortment of PK parameters VM, KM, K, Cl used in differentiating the  **EpoR**  population in newborn and adult sheep.  Ref: 12750427 J Pharmacol Exp Ther< MedlineTA>J Pharmacol Exp Ther, 2003
- Our results suggest that increased expression of  **Epo**  and  **EpoR**  may play a significant role in cervical carcinogenesis and tumor progression.  Ref: 12759237 Am J Pathol, 2003
